# Effect of inhaled interferon-β1a on SARS-CoV-2 diversity and evolution

**DOI:** 10.1128/spectrum.00541-26

**Published:** 2026-05-18

**Authors:** Gregory E. Edelstein, Tatum N. Sass, Rinki Deo, Owen T. Glover, Julie Boucau, Prasanna Jaganathan, Kara W. Chew, Mark J. Giganti, Michael D. Hughes, Carlee Moser, Arzhang Cyrus Javan, Courtney V. Fletcher, Caitlyn McCarthy, David A. Wohl, Eric S. Daar, Joseph J. Eron, Judith S. Currier, Upinder Singh, Davey M. Smith, William Fischer, Amy K. Barczak, Manish C. Choudhary, Jonathan Z. Li, for the ACTIV-2/A5401 Study Team

**Affiliations:** 1 Department of Medicine, Brigham and Women's Hospital 370908, Boston, Massachusetts, USA; 2 Ragon Institute of Mass General Brigham, Cambridge, Massachusetts, USA; 3 Department of Medicine, Stanford University School of Medicine 196258, Stanford, California, USA; 4 Department of Medicine, David Geffen School of Medicine at University of California, Los Angeles 12222, Los Angeles, California, USA; 5 Harvard T.H. Chan School of Public Health 1857 , Boston, Massachusetts, USA; 6 National Institutes of Health 2511, Bethesda, Maryland, USA; 7 College of Pharmacy, University of Nebraska Medical Center 15519, Omaha, Nebraska, USA; 8 Department of Medicine, University of North Carolina at Chapel Hill School of Medicine 214908, Chapel Hill, North Carolina, USA; 9 Lundquist Institute at Harbor-UCLA Medical Center 21640, Torrance, California, USA; 10 Department of Medicine, University of Carolina, San Diego, La Jolla, California, USA; Barnard College, Columbia University Biology, New York, New York, USA

**Keywords:** SARS-CoV-2, antiviral agents, interferons, SARS-CoV-2 evolution, viral diversity

## Abstract

Interferon resistance has been implicated in SARS-CoV-2 escape from innate immunity, but exogenous interferon’s impact on viral evolution and diversity is unknown. SNG001, an inhaled interferon-β1a treatment, was evaluated in the ACTIV-2/A5401 randomized controlled trial of therapeutics for COVID-19. We measured viral kinetics and performed whole-genome sequencing on longitudinal nasal swabs collected from ACTIV-2 participants who received either SNG001 or placebo to assess viral sequence diversity. No difference in nasal viral load decay was detected between study arms when stratifying by SARS-CoV-2 variant or by viral culture conversion. Compared to placebo participants, the SNG001-treated participants displayed significantly lower nonsynonymous amino acid average pairwise distance, indicating lower sequence diversity. Similarly, SNG001-treated individuals also developed numerically fewer nonsynonymous mutations during their infection in ORF1a, ORF1b, Spike, and Nucleocapsid. No specific emerging SARS-CoV-2 nonsynonymous amino acid changes indicating signatures of viral escape were enriched in those receiving SNG001. These *in vivo* data provide an intriguing signal that exogenous interferon-β1a may restrict SARS-CoV-2 viral diversity and add to growing evidence that interferon levels play a critical role in antiviral responses during COVID-19.

**IMPORTANCE:**

SARS-CoV-2 encodes several genes which can antagonize the interferon signaling cascade, preventing it from activating antiviral responses and thereby facilitating viral establishment and dissemination. It is unknown how the administration of exogenous interferon might affect viral evolution and immune escape. ACTIV-2/A5401 represents a unique opportunity to study the virologic effects of interferon treatment in a rigorous randomized, placebo-controlled clinical trial setting. Our characterization of longitudinal nasal samples shows that interferon-treated individuals had lower viral diversity and no evidence of viral escape mutations.

**CLINICAL TRIALS:**

This study is registered with ClinicalTrials.gov as NCT04518410.

## INTRODUCTION

SARS-CoV-2 infection is known to suppress the host interferon (IFN) response, a critical component of innate immunity that induces antiviral responses (1, 2). Evidence suggests at least 16 SARS-CoV-2 proteins antagonize the IFN signaling cascade, with common mechanisms aimed at either blocking the production of IFN by inhibiting IRF3 phosphorylation and nuclear translocation or by preventing the transcription of IFN-stimulated genes by preventing STAT1/STAT2 phosphorylation (2–5). Thus, SARS-CoV-2 appears to prevent both the activation and execution of the IFN-mediated antiviral response. Similarly, clinical studies early during the pandemic indicated that a deficient IFN response might be implicated in progression to severe COVID-19 disease. Individuals who developed life-threatening COVID-19 pneumonia were observed to more commonly have autoantibodies to type I IFNs (6) or harbor genetic defects in type I IFN receptors (7). Additionally, type I IFNs, particularly IFN-β, display potent *in vitro* antiviral activity against SARS-CoV-2 (8).

Despite the critical role that interferon plays in host antiviral immune responses, little is known about the impact of interferon responses on SARS-CoV-2 viral sequence diversity and evolution. As previously described (2–5), several SARS-CoV-2 genes interact with the IFN pathway, so mutations in response to interferon could occur in multiple genes, in comparison to previous antivirals that target a specific viral process and its associated gene, such as nirmatrelvir and Mpro/nsp5 (9, 10) or remdesivir and RdRp/nsp12 (9, 11). Adding to this complexity is that IFN’s mechanism of action also bolsters the host immune response signaling (12, 13) in addition to directly exerting effects on the virus itself. Lastly, phenotypic IFN-resistance has tended to increase with each newly emerging SARS-CoV-2 variant (8, 14, 15), so some variants may be less susceptible to IFN than others.

Given the antiviral activity of IFN *in vitro* (8), as well as *in vitro* and *in vivo* data on SARS-CoV-2 IFN antagonism (1–5), administration of exogenous IFN to bolster the host response was explored as a promising therapeutic treatment for COVID-19. SNG001 is an orally inhaled, type-I recombinant IFN-β1a therapeutic that was previously reported to lead to faster recovery in hospitalized patients with COVID-19 in a phase II study (16).

The ACTIV-2/A5401 randomized placebo-controlled trial (clinical trials.gov NCT04518410) evaluated SNG001 given for 14 days in outpatients with COVID-19 to evaluate its impact on disease progression (17). Although SNG001 did not accelerate nasal viral RNA decay in the ACTIV-2 trial, there was a trend toward decreased hospitalizations in the treated arm compared with placebo, all of which were due to COVID-19 pneumonia (17). Importantly, ACTIV-2 represents a unique opportunity to study the virologic effects of exogenous interferon exposure in a rigorous randomized, placebo-controlled clinical trial setting.

Even among virus-directed antivirals, a wide range of viral genetic changes have been observed in response to their use. For instance, we have previously reported on viral evolution in those receiving antiviral treatment, such as bamlanivimab (18), as well as nirmatrelvir and remdesivir (9). These data and others demonstrate the variety of viral evolutionary patterns that may occur in response to antiviral therapy. Here, we perform whole-genome sequencing of nasal swabs collected in the ACTIV-2 trial of SNG001 and perform sequence analysis to characterize SARS-CoV-2 evolution and diversity in response to exogenous IFN treatment.

## MATERIALS AND METHODS

### ACTIV-2 design

The study population included 220 adult (≥18 years old) outpatients with confirmed COVID-19 enrolled in a phase II assessment of SNG001 in ACTIV-2, a randomized, placebo-controlled, adaptive platform clinical trial (NCT04518410). Participants were randomized to receive either SNG001 or placebo from February to August 2021 (17). Placebo recipients were pooled from those designated to receive an inhaled, SNG001-specific placebo, as well as from those receiving placebos for different investigational agents in the platform trial. Key inclusion criteria included entry into the study within 10 days from diagnosis or symptom onset and the presence of COVID-19 symptoms during the 24 h prior to study entry. The study protocol was approved by a central institutional review board (IRB), Advarra, with additional local IRB approval if required by participating sites. All participants provided written informed consent. Additional details are published elsewhere (17).

Participants self-administered either SNG001 (two syringes of 0.65 mL of recombinant IFN-β1a at a concentration of 12 MIU/mL) or orally inhaled placebo (two syringes of 0.65 mL placebo solution) via Aerogen Ultra Nebulizer device for approximately 4 min/day for 14 days. Previous studies estimate the IFN-β1a dosage delivered to the lungs at 3.8 to 5 MIU (16, 19). Participants in the shared placebo arm with another ACTIV-2 agent received either oral or intravenous placebos. Nasopharyngeal (NP) and nasal (NAS) swabs were collected by study staff at days 0 (before any intervention was administered), 3, 7, 14, and 28 (17).

### Virologic methods

#### Viral load testing

SARS-CoV-2 quantitative RNA viral load was performed at a central laboratory (University of Washington) using the Abbott m2000sp/rt platform in conjunction with Abbott SARS-CoV-2 calibration standards to correlate cycle threshold and RNA copies per mL. Assay validation determined the lower limit of quantification of <2 log_10_ RNA copies/mL and the upper limit of quantification of 8 log_10_ RNA copies/mL (17, 20). Results above the upper limit of quantification were repeated with sample dilution.

#### Viral culture

Viral culture was performed in the BSL3 laboratory of the Ragon Institute of Mass General Brigham, Harvard, and MIT as described previously (21–23). Viral transport media (VTM) from NP swabs was filtered through 0.45-μm filters and used to inoculate Vero-hAce2-hTMPRSS2 (BEI Resources) seeded in 96 well plates in media supplemented with polybrene (Santa Cruz Biotechnology). The plates were spun for 1 h at 2,000 *g* and then incubated at 37°C and 5% CO_2_. After 7 days, plates were evaluated for cytopathic effects on a brightfield microscope and median tissue culture infectious dose (TCID_50_/mL) titers were calculated using the Reed-Muench method.

#### Viral RNA extraction

For samples with quantifiable viral loads (i.e., ≥2.0 log_10_ RNA copies/mL), SARS-CoV-2 viral RNA was extracted from NP or NAS swab VTM as previously described (24). Briefly, 1,000 µLs of VTM was used for Trizol-LS extraction (Invitrogen), after which pelleted RNA was resuspended in 22 µLs of diethylpyrocarbonate-treated water (ThermoFisher Scientific). cDNA synthesis was performed using Superscript IV reverse transcriptase (Invitrogen) following the manufacturer’s instructions and as reported elsewhere (9).

#### SARS-CoV-2 whole-genome sequencing

Since exogenous IFN could affect changes across multiple genes throughout the viral genome, we utilized whole-genome sequencing (WGS). The SARS-CoV-2 genome was amplified using NEBNext (New England Biolabs) multiplexed Varskip primer pools 1 & 2 designed with Primal Scheme generating 550-bp tiling amplicons. PCR products from primer pools 1 & 2 were pooled together for each sample separately prior to library preparation. Next-generation sequencing was performed using the Illumina MiSeq platform. Raw sequencing data were analyzed using Stanford University’s Coronavirus Antiviral & Resistance Database (CoV-RDB) (25). Initial alignment of input FASTQ sequences to the Wuhan-Hu-1 reference was performed using MiniMap2 (version 2.22) within the CodFreq pipeline (https://github.com/hivdb/codfreq). The resulting aligned SAM file from MiniMap2 was then converted to a CodFreq file using an in-house Python script leveraging the PySam library (version 0.18.0) and subsequently subjected to further analysis with CoV-RDB (25).

#### Mutation detection and SARS-CoV-2 variant call

At the codon level, amino acid variants were called at a threshold of ≥10% of the total sequenced viral population, regardless of the sample viral load. This threshold is more conservative than that of our previous SARS-CoV-2 work sequencing singular genes (9) to account for the lower input volume of viral RNA used for WGS. SARS-COV-2 variant calling was performed using Nextclade (26) and CoV-RDB (25) for participants with successful sequencing at the baseline timepoint (i.e., study day 0) from an NP swab with quantifiable viral load.

### Longitudinal virologic analysis

The viral kinetics analysis included all participants with successful sequencing and quantifiable NP viral load at the baseline sample, as well as a measured NP viral load (either quantifiable or unquantifiable) on at least one follow-up timepoint. To more directly measure replicating virus, we also compared viral culture positivity at baseline and day 3 for a subset of these participants that had baseline quantifiable viral load ≥6 log_10_ RNA copies/mL and available samples.

### Viral diversity analyses

Inclusion in the viral diversity analyses required a participant to have both successful sequencing and a quantifiable NP viral load at baseline and at least one follow-up timepoint. Each participant’s consensus sequence at baseline served as the reference for comparisons of the later timepoints. Amino acid variants present at 100% frequency of the total sequenced viral population at all timepoints were considered indicative of lineage-defining mutations and were excluded from these analyses.

#### Amino acid average pairwise distance

Genetic diversity between multiple sequences (baseline and at least one follow-up timepoint) of an individual was assessed by within-group average pairwise distance (APD) in MEGA 11 (27) at the amino acid level using the consensus sequence as described previously (28). For each participant, APD was normalized to the number of days (APD per day) at which the last SARS-CoV-2 sequence during infection was collected, calculated from the baseline sequence as day 0.

#### Synonymous to nonsynonymous mutation comparisons

We performed two additional analyses of synonymous and nonsynonymous sequence variation using MEGA 11 (27): synonymous APD/day and the ratio of nonsynonymous to synonymous substitutions (d*N*/d*S*). Within-group synonymous APD per day was calculated in MEGA 11 using the distance estimation framework under the synonymous/nonsynonymous substitution model, with the Nei-Gojobori method (Jukes-Cantor correction), synonymous substitutions only, and pairwise deletion for gaps and missing data. We also calculated d*N*/d*S* for each participant over the course of infection. A *Z*-test of neutral selection was then used to identify participants whose SARS-CoV-2 sequences showed evidence of deviation from neutral evolution.

#### Emergent amino acid polymorphisms

Emergent amino acid polymorphisms were defined as nonsynonymous mutations that were not present in a participant’s baseline sequence and were detected in later follow-up samples. We investigated the count of mutations per each SARS-CoV-2 gene, normalized by the gene length in kilobases (Kb). Additionally, we investigated whether specific nonsynonymous mutations emerged significantly more frequently in either the SNG001 or placebo group participants compared to the other group.

### Statistical methods

Categorical variables were described using frequencies and percentages, and differences between groups were assessed using Fisher’s exact test or chi-square test where appropriate. Continuous variables were summarized as medians with interquartile ranges and compared using the Wilcoxon rank-sum test. Statistical analyses were conducted using R software (version 4.3.0). All tests were two-tailed, with statistical significance *α* = 0.05; analyses were not adjusted for multiple comparisons.

Analyses were also stratified by SARS-COV-2 variant of infection (Alpha, Delta, or Other), or by timing from symptom onset to the first study nasal swab, either early initiation (<5 days) or late initiation (≥5 days). These stratifications, respectively, allowed us to assess for the presence of variant-specific effects of IFN on viral load or mutations (8, 14, 15), as well as if early-administration of SNG001 is more effective, a pattern that has been observed for other SARS-CoV-2 antivirals (29, 30).

## RESULTS

### Participant characteristics and viral kinetics

**Fig 1:**
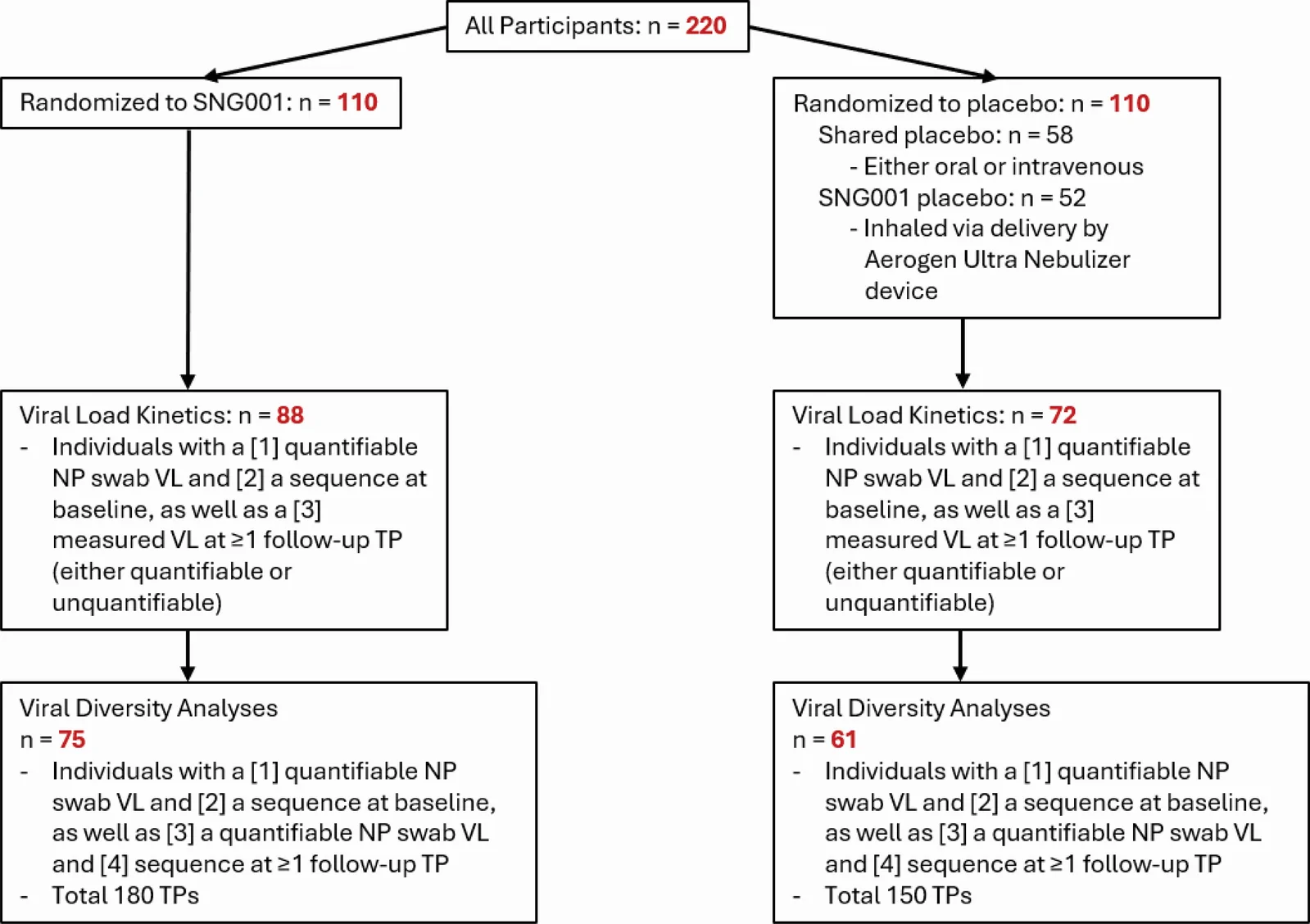
Participant flow diagram. Each arm enrolled 110 participants, with 52 in the placebo group receiving SNG001-designated placebo via nebulizer, and 58 receiving placebo for another ACTIV-2 agent (see Materials and Methods). SARS-CoV-2 sequencing and subsequent variant call were performed for each timepoint with a quantifiable nasopharyngeal viral load. Participants with a quantifiable nasopharyngeal viral load at baseline and at least one follow-up viral load (quantifiable or unquantifiable) were included in the viral kinetics analysis. The viral diversity analyses included participants with quantifiable viral load and sequences at baseline and at least one follow-up timepoint. Abbreviations: VL, viral load; NP, nasopharyngeal; PID, participant; TP, timepoint.

Overall, 160 participants (SNG001 *n* = 88; placebo *n* = 72) had a quantifiable baseline viral load which could be used to generate a variant call and were included in the viral load kinetics analysis (Fig. 1). Participant demographics were similar in each arm (Table 1); median age was 40 years, 54% were female sex, and 10% were Black. For COVID-19-related characteristics, 44% of participants met our criteria for early study initiation, collecting their first study swab <5 days from symptom onset, and 16% were considered at high-risk for progression to severe COVID-19 per the study protocol definition. The median baseline viral load was 5.1 log_10_ RNA copies/mL (Table 1).

**TABLE 1:** Cohort characteristics

	SNG001 (*n* = 88)	Placebo (*n* = 72)	Overall (*n* = 160)	*P*-value
Age (years), Median (Q1, Q3)	41 (32, 49)	40 (35, 51)	40 (33, 50)	0.75^*b*^
Sex, *n* (%)				0.15^*c*^
Female	52 (59)	34 (47)	86 (54)	
Male	36 (41)	38 (53)	74 (46)	
Race, *n* (%)				0.36^*d*^
White	68 (77.3)	56 (77.8)	124 (77.5)	
Black	11 (12.5)	5 (6.9)	16 (10.0)	
Other/Multiple/Missing	9 (10.2)	11 (15.3)	20 (12.5)	
Ethnicity, *n* (%)				0.27^*c*^
Hispanic or Latino	42 (48)	41 (57)	83 (52)	
Not Hispanic or Latino	46 (52)	31 (43)	77 (48)	
Risk category for progression to severe COVID-19, *n* (%)				1.00^*c*^
Higher	14 (16)	12 (17)	26 (16)	
Lower	74 (84)	60 (83)	134 (84)	
Days from symptom onset to study day 0, *n* (%)				0.11^*c*^
Early^*a*^ (<5 days)	34 (39)	37 (51)	71 (44)	
Late^*a*^ (≥5 days)	54 (61)	35 (49)	89 (56)	
SARS-CoV-2 Variant, *n* (%)				0.22^*d*^
Alpha	26 (30)	19 (26)	45 (28)	
Delta	40 (45)	26 (36)	66 (41)	
Other (non-Alpha/non-Delta)	22 (25)	27 (38)	49 (31)	
Baseline nasopharyngeal swab viral load (log_10_ RNA copies/mL), Median (Q1, Q3)	5.3 (4.3, 6.3)	5.6 (4.4, 6.9)	5.3 (4.3, 6.4)	0.23^*b*^

^*a*^ Early and Late were defined relative to the median days from symptom onset to study day 0 (i.e., 5 days) for the entire cohort, as previously described (17).

^*b*^ Wilcoxon rank sum.

^*c*^ Fisher’s exact test.

^*d*^ Chi-square test.

**Fig 2:**
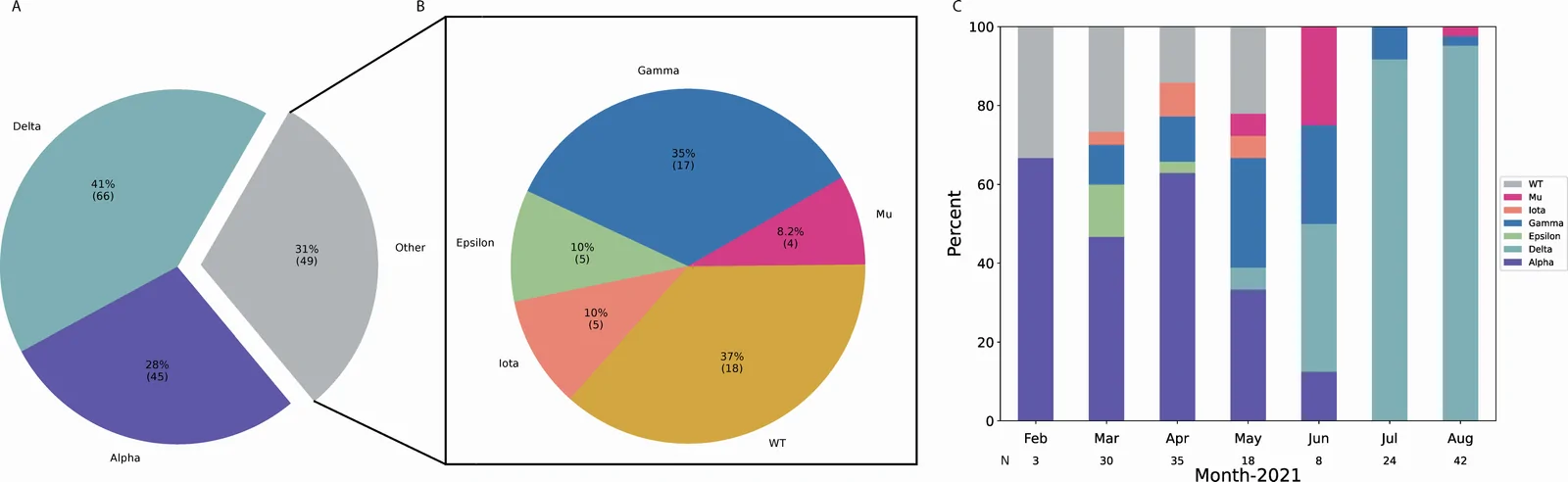
SARS-CoV-2 variant distribution of the SNG001 Phase 2 ACTIV-2 trial during the enrollment period. (**A**) Proportion of the three most common variant categories, Alpha, Delta, and all Other variants, in the trial. (**B**) Proportions of the SARS-CoV-2 variants that comprise the “Other” variant category. (**C**) Variant distribution and the number of participants enrolling in the SNG001 trial by month (February to August 2021).

The variant distributions were similar across arms (Table 1). The Alpha, Delta, and Other SARS-CoV-2 variants comprised 28%, 41%, and 31% of infections, respectively (Fig. 2A). The most common variants among the “Other” category were wild-type (37%) and Gamma (35%) (Fig. 2B). The frequency of variants in the trial population changed over the enrollment period (Fig. 2C).

**Fig 3:**
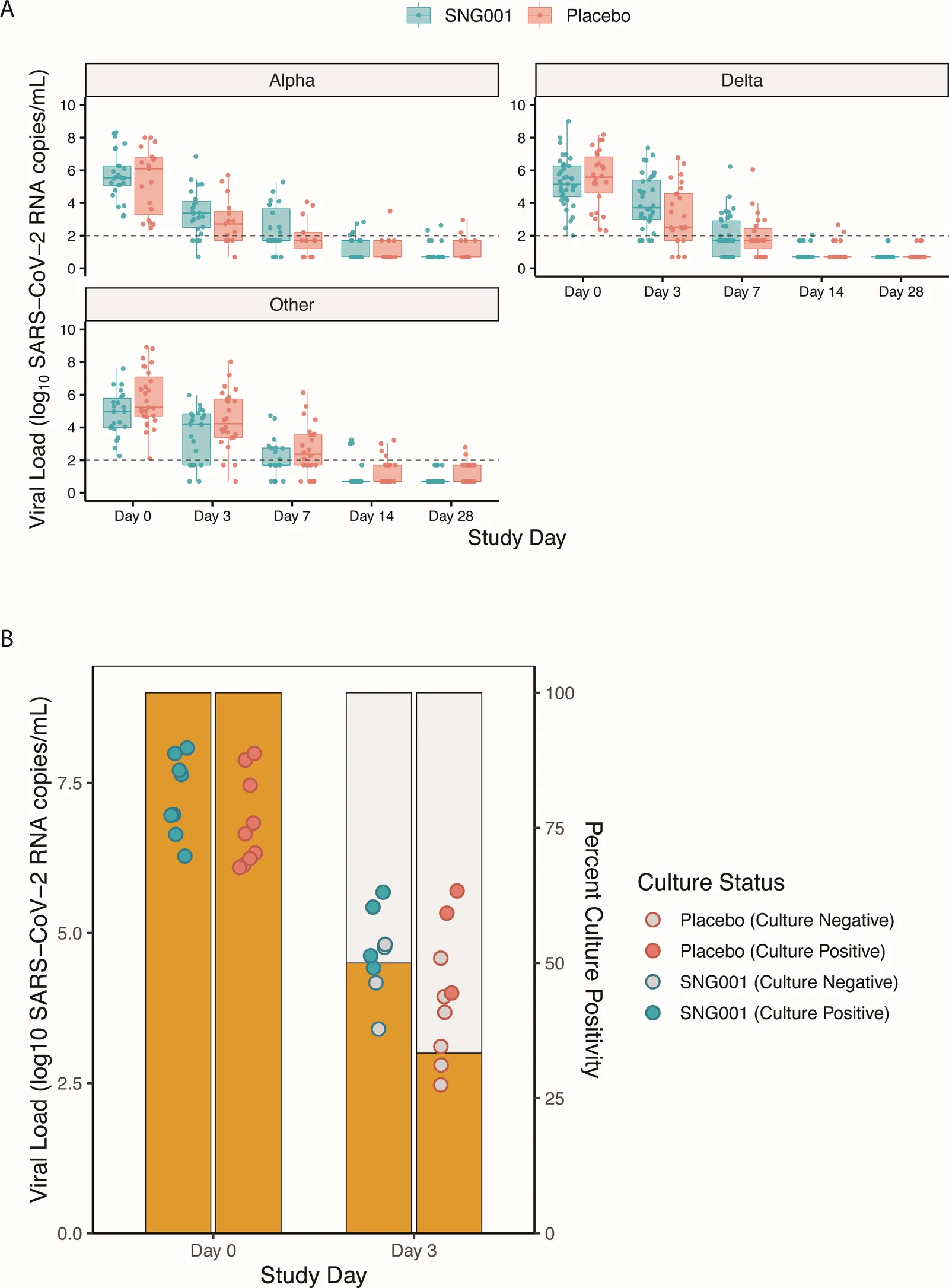
Viral kinetics analyses demonstrate that inhaled interferon-β1a does not accelerate viral RNA decay when stratified by SARS-CoV-2 variant or by viral culture conversion in a subset of participants. (**A**) Viral load analysis stratified by SARS-CoV-2 variant category. At each timepoint, the distributions of viral loads of the SNG001 and placebo groups are compared using two-sided Wilcoxon rank sum tests. The lower limit of quantification (<2.0 log_10_ RNA copies/mL) is indicated by the dashed line; viral loads detected below this threshold are imputed as 1.7 log_10_ RNA copies/mL, while undetectable viral loads are imputed as 0.7 log_10_ RNA copies/mL. Box plots depict median, interquartile range, and range. (**B**) Viral culture positivity in a subset of 17 participants (8 SNG001-treated, 9 placebo) with day 0 SARS-CoV-2 RNA levels ≥ 6 log_10_ RNA copies/mL and available sample at day 3. The percentage of culture positive samples is depicted using orange bars, and the circles indicate RNA viral load with closed and open circles representing culture-positive and culture-negative samples, respectively.

In 160 participants with quantifiable NP swab viral load at baseline, we did not identify any timepoints in which the treatment group had significantly lower viral load than the placebo group when stratified by SARS-CoV-2 variant of infection (Fig. 3A). These results are similar to the viral load kinetic results of the primary analysis, which did not stratify by variant (17). In addition, we performed viral culture in a subset of 17 participants (8 SNG001-treated, 9 placebo) with baseline SARS-CoV-2 RNA levels ≥ 6 log_10_ RNA copies/mL and available NP swab at day 3. Percent viral culture positivity was similar between groups at baseline (both 100%) and at day 3 (SNG001 vs placebo, 50% vs 33%, *P* = 0.64, Fig. 3B), confirming that SNG001 treatment had no significant effect on the shedding of infectious virus.

### Within-group average pairwise distance

The viral sequence diversity analyses utilized a subset of 136 individuals (SNG001 vs placebo, *n* = 75 vs *n* = 61) with quantifiable NP viral load and successful sequencing at both baseline and at ≥1 follow up timepoint. The median number of sequenced timepoints per participant were similar between groups (SNG001 vs placebo, 2.47 vs 2.46 timepoints, *P* = 0.67, Wilcoxon rank-sum) (Fig. 1). This subset of participants was demographically similar between arms, with the exception that more participants in the placebo group (*n* = 38, 62%) entered the study early (<5 days from symptom onset) compared to the SNG001 group (*n* = 33, 44%, *P* = 0.04) (Table S1).

**Fig 4:**
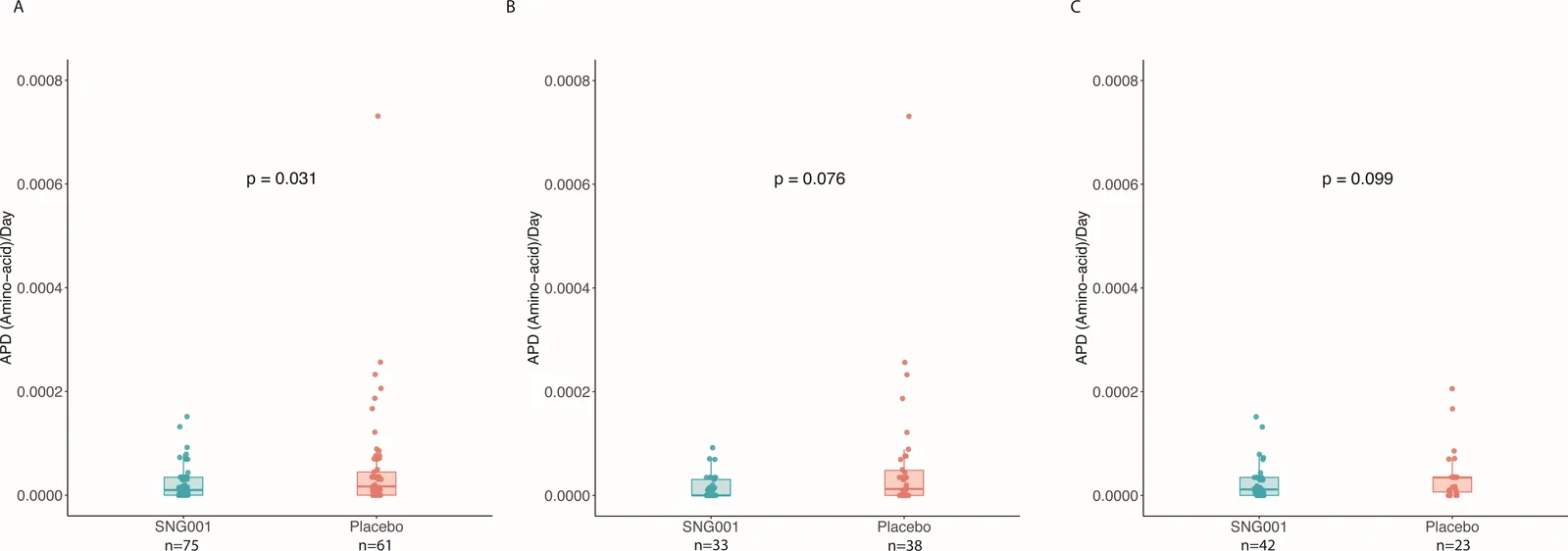
Individuals treated with inhaled interferon-β1a have significantly lower within-group amino acid diversity than the placebo arm. Amino acid average pairwise distance normalized to the day of last collected sequence (APD/day) for (**A**) the entire SNG001 and placebo arms, and when stratified by days from symptom onset to baseline nasal swab, either early (<5 days, **B**) or late (≥5 days, **C**). Distributions of average pairwise distance of the SNG001 and placebo arms are compared using two-sided Wilcoxon rank sum tests. Box plots depict median, interquartile range, and range.

**Fig 5:**
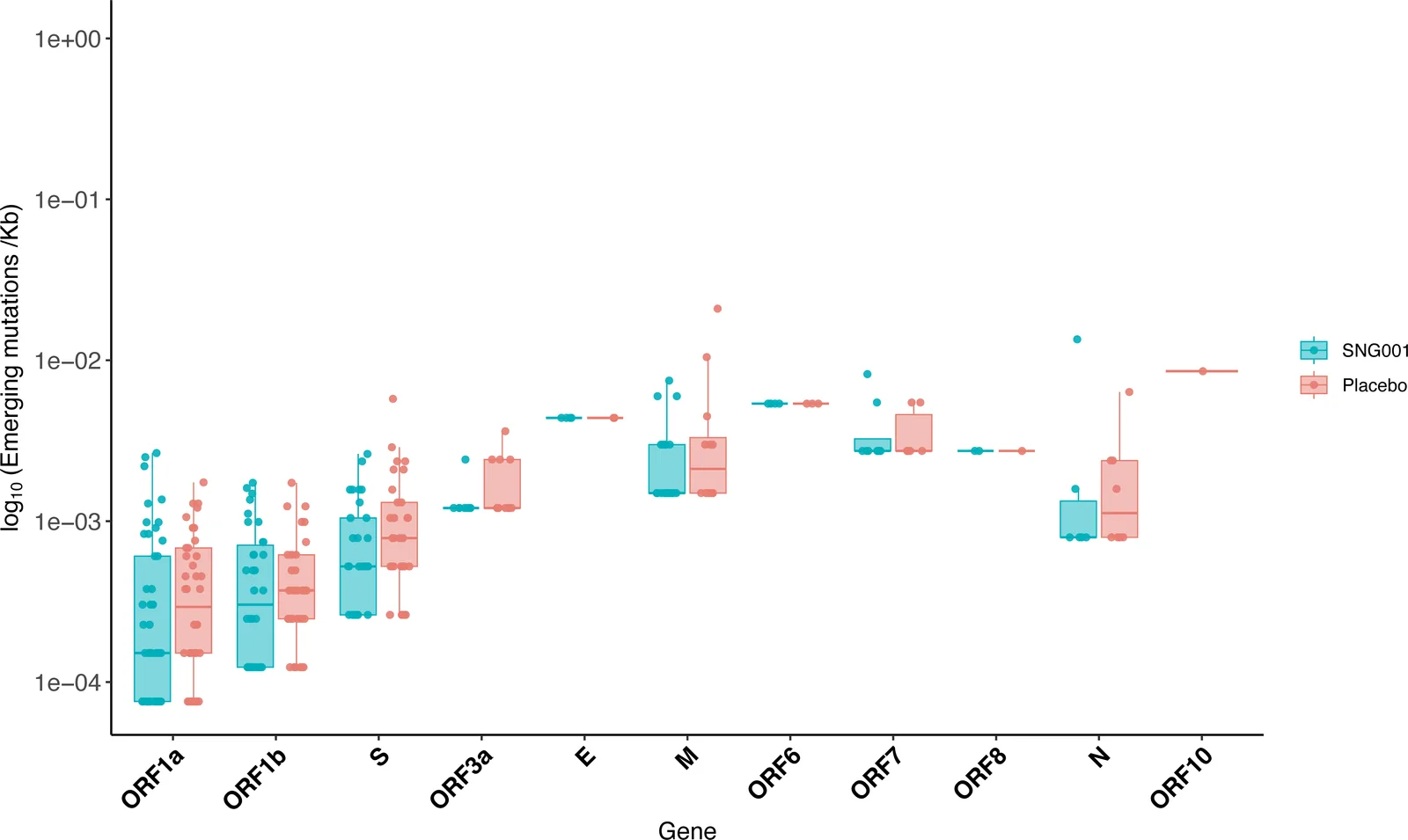
Individuals treated with inhaled interferon-β1a developed numerically fewer emerging nonsynonymous amino acid changes in SARS-CoV-2 genes compared to untreated individuals. Counts of emergent (not present in the baseline sequence and emerged at later timepoints) nonsynonymous amino acid changes in each SARS-CoV-2 gene, normalized to gene length, depicted with a log_10_ scale. Distributions of the counts of emerging mutations in the SNG001 and placebo groups are compared using two-sided Wilcoxon rank sum tests. Box plots depict median, interquartile range, and range. Abbreviations: Kb, kilobase; ORF, open reading frame; S, spike; E, envelope; M, membrane; N, nucleocapsid.

Inhaled interferon recipients had significantly lower within-group amino acid diversity (SNG001 vs placebo, median APD/day 9.9 × 10^−6^ vs 16.9 × 10^−6^, *P* = 0.031) (Fig. 4A). Lower APD per day in the SNG001 group is indicative of fewer changes in the viral sequence over multiple timepoints during infection. The removal of an outlier with a high APD/day in the placebo group did not significantly affect this pattern (*P* = 0.044, data not shown). A trend of lower viral diversity with inhaled interferon was similarly observed in participants who collected their first NP swab less than 5 days (SNG001 vs placebo, median APD/day 0.0 vs 1.3 × 10^−5^, *P* = 0.076) (Fig. 4B) or 5 or more days from symptom onset (SNG001 vs placebo, median APD/day 1.2 × 10^−5^ vs 3.5 × 10^−5^, *P* = 0.099) (Fig. 4C), albeit non-significantly.

Using the same set of 136 participants, we additionally examined synonymous APD/day (Fig. S1A) and d*N*/d*S* ratio (Fig. S1B) although neither comparison revealed significant differences between the groups (all *P* > 0.34). Only two participants, one in each arm, had SARS-CoV-2 whole-genome sequences showing significant evidence of positive evolution during infection (d*N*/d*S* > 2.02; *P* < 0.045, *Z*-test for neutral selection, Fig. S1B) during their infection.

### Emergent nonsynonymous amino acid mutations

We next evaluated the frequency of emergent polymorphisms across the SARS-CoV-2 genome to determine potential signatures of interferon escape. Individuals receiving SNG001 had fewer emergent nonsynonymous mutations for four genes when normalized to gene length: ORF1a (SNG001 vs placebo, median 1.5 × 10^−4^ vs 3.0 × 10^−4^ mutations per Kb, fold change [SNG001/placebo] = 0.50), ORF1b (3.1 × 10^−4^ vs 3.7 × 10^−4^ per Kb, fold change = 0.83), Nucleocapsid (7.9 × 10^−4^ vs 11.9 × 10^−4^ per Kb, fold change = 0.67), and Spike (5.2 × 10^−4^ vs 7.8 × 10^−4^ per Kb, fold change = 0.67), although these comparisons did not reach statistical significance (Fig. 5). For all other genes, the median number of nonsynonymous changes was similar between groups. No emerging mutations were detected in ORF10 of SNG001-treated participants.

We did not detect any amino acid signatures of interferon-associated viral mutations. Although 48 mutations were observed to emerge in multiple (≥3) participants at ≥10% of the viral population in both groups (Table S2), there were only 4 emergent amino acid mutations that were significantly different between the 2 arms, and all 4 were more common in the placebo arm in unadjusted comparisons (Fig. S2). These mutations represented a small proportion of the sequenced viral population (all less than 39%), are of unknown functional significance, and appear infrequently in sequence repositories like GISAID (31).

## DISCUSSION

In this analysis of participant nasal swab samples collected in the ACTIV-2 trial, we found that an inhaled IFN-β1a therapy, SNG001, did not lead to changes in SARS-CoV-2 viral RNA kinetics or viral culture conversion in individuals with acute COVID-19. Among the 136 participants with sequences at baseline and at least one follow-up timepoint, we detected a pattern of lower viral diversity in the SNG001-treated group as reflected by lower amino acid average pairwise distance. This pattern was observed in the treated arm and did not differ meaningfully when stratified by early vs late treatment initiation or when comparing rates of synonymous to nonsynonymous mutations. We also observed numerically fewer nonsynonymous amino acid mutations emerging during SARS-CoV-2 infection in the ORF1a, ORF1b, Spike, and Nucleocapsid genes of SNG001-treated participants compared to placebo participants. Lastly, we did not detect any signatures of interferon escape, with the only emergent mutations that significantly differed between the two arms being more prevalent in the placebo group.

Our viral diversity analyses of APD and emerging nonsynonymous mutations suggest that IFN-β1a treatment may dampen viral diversification and escape in SARS-CoV-2. Interestingly, we find that this pattern is not driven by early treatment administration in our study, contrasting with previous *in vitro* and *in vivo* studies of IFN treatment suggest that early exposure to both type I (32–35) and type III (32, 35, 36) IFNs may be crucial for its efficacy. Hypotheses for timing-dependent benefits of early exposure to IFN include early activation of interferon-stimulated genes (ISGs) and IFN-coordinated activation of host cellular immunity. Some ISGs, such as IFITMs (37–39), LY6E (40, 41), and CH25H (42), inhibit viral entry by Spike-mediated membrane fusion, while others like BST2 inhibit viral egress (43), which could reduce widespread viral dissemination if activated early by endogenous or exogenous IFN. Type I IFNs also promote proliferation of natural killer cells and enhance their cell-killing capacity, support the differentiation of monocytes into antigen-presenting dendritic cells, and aid in the chemotactic migration of dendritic cells to sites of infection (12, 13). In a human challenge study of SARS-CoV-2, Lindeboom and colleagues (44) observed increased immune cell infiltration in nasopharynx epithelial cells 1 day after inoculation in participants that developed only a transient infection, compared to 5 days after inoculation in participants that developed sustained infections. This suggests that early immune responses, which may be aided by early IFN upregulation, are important in limiting the spread of viral infection, whereas late responses may be ineffective. However, the optimal window for therapeutic benefit of exogenous IFN administration remains largely undetermined (45) and may be unrelated to the effect we observe here of reduced viral diversity with SNG001 in a timing-independent manner.

A mechanistic explanation for how IFN restricts SARS-CoV-2 diversity is not fully clear and may be multifactorial. One potential explanation for the lower viral diversity with IFN exposure is that administration of exogenous IFN can activate ISGs that halt the virus in a pre-replication step (37–43, 46) or may directly interfere with viral polymerases (47–49) leading to lower diversity since fewer viral genomes are generated. For example, the ISG ZAP interferes with the production of SARS-CoV-2 polyprotein 1a/b (47) which encodes critical components of viral replication machinery and also degrades SARS-CoV-2 viral RNA by binding to CpG-rich regions and recruiting cellular proteins (48). Another ISG, TRIM22, encodes an E3 ubiquitin ligase that polyubiquitinates nsp8, a component of the replication-transcription complex that recognizes viral RNA, targeting it for proteasomal degradation (49). However, this would also be expected to decrease viral replication and viral load, which was not observed in this study. Notably, the ACTIV-2 trial only collected nasal swabs, which sample the upper respiratory tract, while SNG001 is orally inhaled into the lungs. Both viral load kinetics (50) and viral polymorphisms (51) can differ between these two compartments. It is possible an unmeasured decline in viral load occurred in the lungs, which may be supported by the trend towards reduced hospitalizations due to COVID-19 pneumonia in the SNG001-treated participants (17). Future studies investigating a link between IFN and reduced viral diversity should sample multiple anatomical compartments.

Another possibility is that IFN-treatment could affect polymerase fidelity to induce fewer mutations, resulting in lower sequence diversity observed in SNG001-treated participants. For example, the ISG TRIM32 binds and SUMOylates the exonuclease domain of nsp14, preventing it from binding to its nsp10 co-factor and efficiently performing proof-reading activity during SARS-CoV-2 replication (52). However, one could expect this decreased fidelity to lead to more mutations, the opposite pattern we observe here. Indeed, prior studies of SARS-CoV engineered to lack nsp14 exonuclease activity resulted in higher mutational burdens and attenuated virulence both *in vitro* (53, 54) and *in vivo* (55). Although there have been reports of mutations that confer increased polymerase fidelity in poliovirus (56) and chikungunya virus (57) without affecting viral replication kinetics, the pattern we observe in the SNG001 trial, these mutations also conferred reduced fitness (56, 57) and altered tissue tropism (56), and, to our knowledge, no similar mutation has been reported for SARS-CoV-2.

Besides the direct-acting effects of ISGs, the four genes for which we observed numerically reduced emerging mutations (ORF1a, ORF1b, Spike, and Nucleocapsid) produce proteins that are substantially immunogenic (58–61), which could also affect viral diversity. One study estimates that Spike, Nucleocapsid, nsp3 and nsp4 (of ORF1a), and nsp12 (of ORF1b) combined account for 56% and 64% of CD8+ and CD4+ T cell reactivity, respectively, in activation assays (61). Since IFN activates and enhances the immune response (12, 13), it is possible that increased immune pressure creates genetic bottleneck effects and/or purifying selection.

Consistent with the APD results, we also detected fewer nonsynonymous emergent mutations in ORF1a, ORF1b, Spike, and Nucleocapsid. Notably, the protein products of each of these four genes antagonize the IFN pathway through some mechanism(s). ORF1a encodes nsp1 through nsp11 and ORF1b encodes nsp12 through 16, some of which antagonize IFN as reviewed by Justo Arevalo and colleagues (3), while Nucleocapsid and Spike IFN-antagonism are well-characterized. The expression of Nucleocapsid protein interferes with the detection of pathogen-associated molecular patterns by the RIG-I receptor (62–64) and also inhibits phosphorylation of STAT1, STAT2, and IRF3 (62, 64, 65). Spike is a major phenotypic determinant of IFN resistance (15, 38, 39) and interacts with and inhibits IRF3 (66) and IRF9 (67), respectively preventing IFN production and transcription of ISGs.

In our previous analyses of anti-SARS-CoV-2 therapeutics, we were able to identify evidence of emerging drug resistance (9, 18). For instance, participants receiving the antiviral monoclonal antibody bamlanivimab developed resistance mutations in Spike that became fixed in the viral population over time (18), while resistance-associated mutations in nsp5 and nsp12 appeared only transiently and at low frequencies in the setting of nirmatrelvir-ritonavir and remdesivir use (9). However, we have not previously observed the pattern of reduced viral diversity that we identified here with SNG001 exposure. This is potentially due to the host-oriented nature of the SNG001 therapy that may impact viral diversification. Together, these data showcase the variety of SARS-CoV-2 evolutionary patterns in response to treatment, which may be mediated by the target of the antiviral (9, 18), fitness costs to the mutation (10), pre-treatment viral burden (18, 68), or host immune responses (28), among other factors.

This study should be interpreted in the context of its limitations. As mentioned, we were unable to sample the lower respiratory tract for viral load or sequencing. Future work should examine multiple anatomical compartments and employ the use of multiple techniques (viral load and viral culture) to robustly investigate patterns in viral replication. We also do not have any data on SNG001 drug levels or its downstream effects on ISGs although both have been studied previously (69). Lastly, we only examined viral sequences, but expression levels of SARS-CoV-2 proteins could also mediate IFN-resistance (14), which would not be revealed by a sequence analysis.

Although inhaled SNG001 did not accelerate nasal viral RNA or culture decay, we identified evidence of decreased viral sequence diversity and potentially less frequent emerging amino acid polymorphisms in those receiving SNG001 compared to placebo. Together, these findings support the hypothesis that exogenous interferon exposure can shape within-host SARS-CoV-2 diversity.

## Data Availability

The authors confirm that all data underlying the findings are fully available. Due to ethical restrictions, study data are available upon request at https://submit.mis.s-3.net/ and will require the written agreement of Advancing Clinical Therapeutics Globally for HIV/AIDS and Other Infections (ACTG) and the manufacturer of the investigational product, Synairgen Research Ltd. Completion of an ACTG Data Use Agreement may be required. The SARS-CoV-2 whole-genome sequence data have been deposited in the NCBI Sequence Read Archive under BioProject accession PRJNA1415307.

## SUPPLEMENTAL MATERIAL

Supplemental figures and tables spectrum.00541-26-s0001.docx Fig. S1 and S2, and Tables S1 and S2.

ASM does not own the copyrights to Supplemental Material that may be linked to, or accessed through, an article. The authors have granted ASM a non-exclusive, world-wide license to publish the Supplemental Material files. Please contact the corresponding author directly for reuse.
