# Supplementary material for: Effect of inhaled interferon-β1a on SARS-CoV-2 diversity and evolution
Source: Microbiol Spectr. 2026 May 18;14(7):e00541-26. doi: 10.1128/spectrum.00541-26 (PMC13339817; doi:10.1128/spectrum.00541-26)

**Supplemental Table 1. Cohort Characteristics of Individuals in the Viral Diversity analyses**

|  | SNG001  (n = 75) | Placebo  (n = 61) | Overall  (n = 136) | P-value |
| --- | --- | --- | --- | --- |
| Age (years), Median (Q1, Q3) | 40 (32, 49) | 41 (34, 48) | 41 (32, 49) | 0.80^b^ |
| Sex, n (%) |  |  |  | 0.06^c^ |
| Female | 46 (61) | 27 (44) | 73 (54) |  |
| Male | 29 (39) | 34 (56) | 63 (46) |  |
| Race, n (%) |  |  |  | 0.60^d^ |
| White | 57 (76.0) | 48 (78.7) | 105 (77.2) |  |
| Black | 10 (13.3) | 5 (8.2) | 15 (11.0) |  |
| Other/Multiple/Missing | 8 (10.7) | 8 (13.1) | 16 (11.8) |  |
| Ethnicity, n (%) |  |  |  | 0.17^c^ |
| Hispanic or Latino | 36 (48) | 37 (61) | 73 (54) |  |
| Not Hispanic or Latino | 39 (52) | 24 (39) | 63 (46) |  |
| Risk category for progression to severe COVID-19, n (%) |  |  |  | 1.00^c^ |
| Higher | 12 (16) | 10 (16) | 22 (16) |  |
| Lower | 63 (84) | 51 (84) | 114 (84) |  |
| Days from symptom onset to study day 0, n (%) |  |  |  | 0.04^c^ |
| Early^a^ (<5 days) | 33 (44) | 38 (62) | 71 (52) |  |
| Late^a^ (≥5 days) | 42 (56) | 23 (38) | 65 (48) |  |
| SARS-CoV-2 Variant, n (%) |  |  |  | 0.11^d^ |
| Alpha | 24 (32) | 16 (26) | 40 (29) |  |
| Delta | 34 (45) | 21 (34) | 55 (40) |  |
| Other (non-Alpha/non-Delta) | 17 (23) | 24 (39) | 41 (30) |  |
| Baseline nasopharyngeal swab viral load (log_10_ RNA copies/mL), Median (Q1, Q3) | 5.5 (4.5, 6.3) | 5.6 (4.7, 7.0) | 5.5 (4.5, 6.6) | 0.32^b^ |

^a^ Early and Late were defined relative to the median days from symptom onset to study day 0

(i.e., 5 days), for the entire cohort, as previously described (17).

^b^ Wilcoxon rank sum.

^c^ Fisher’s exact test.

^d^ Chi-square test.

**Supplemental Table 2.** Emergent nonsynonymous mutations occurring in three or more participants across both arms in the study. Abbreviations: M, membrane; nsp, non-structural protein; PLpro, papain-like protease; RdRp, RNA-dependent RNA polymerase; S, spike.

| Gene | Mutation | Total N with Mutation | SNG001 N with Mutation | Placebo N with Mutation | SNG001 Percent with Mutation^a^ | Placebo Percent with Mutation^b^ | Percent Difference between Arms (Absolute Value) | P-value |
| --- | --- | --- | --- | --- | --- | --- | --- | --- |
| M | A2T | 16 | 10 | 6 | 13.3 | 9.8 | 3.5 | 0.60 |
| M | A2P | 8 | 5 | 3 | 6.7 | 4.9 | 1.7 | 0.73 |
| nsp1 | V84del | 4 | 3 | 1 | 4.0 | 1.6 | 2.4 | 0.63 |
| nsp1 | M85del | 4 | 2 | 2 | 2.7 | 3.3 | 0.6 | 1.00 |
| nsp1 | V86del | 3 | 3 | 0 | 4.0 | 0.0 | 4.0 | 0.25 |
| nsp2 | A419P | 11 | 7 | 4 | 9.3 | 6.6 | 2.8 | 0.75 |
| nsp2 | A419V | 5 | 2 | 3 | 2.7 | 4.9 | 2.3 | 0.66 |
| nsp2 | K347T | 3 | 0 | 3 | 0.0 | 4.9 | 4.9 | 0.09 |
| nsp2 | M418T | 3 | 1 | 2 | 1.3 | 3.3 | 1.9 | 0.59 |
| nsp2 | I484S | 3 | 3 | 0 | 4.0 | 0.0 | 4.0 | 0.25 |
| nsp4 | Y182G | 11 | 6 | 5 | 8.0 | 8.2 | 0.2 | 1.00 |
| nsp4 | A446V | 3 | 2 | 1 | 2.7 | 1.6 | 1.0 | 1.00 |
| nsp6 | K4R | 3 | 0 | 3 | 0.0 | 4.9 | 4.9 | 0.09 |
| nsp8 | S8F | 3 | 1 | 2 | 1.3 | 3.3 | 1.9 | 0.59 |
| nsp14 | A482V | 5 | 1 | 4 | 1.3 | 6.6 | 5.2 | 0.17 |
| nsp16 | D179V | 14 | 7 | 7 | 9.3 | 11.5 | 2.1 | 0.78 |
| nsp16 | A178V | 6 | 4 | 2 | 5.3 | 3.3 | 2.1 | 0.69 |
| nsp16 | D179G | 4 | 3 | 1 | 4.0 | 1.6 | 2.4 | 0.63 |
| nsp16 | D108E | 3 | 0 | 3 | 0.0 | 4.9 | 4.9 | 0.09 |
| nsp16 | D179A | 3 | 1 | 2 | 1.3 | 3.3 | 1.9 | 0.59 |
| PLpro | D471G | 6 | 3 | 3 | 4.0 | 4.9 | 0.9 | 1.00 |
| PLpro | E1025D | 4 | 4 | 0 | 5.3 | 0.0 | 5.3 | 0.13 |
| PLpro | T678P | 3 | 2 | 1 | 2.7 | 1.6 | 1.0 | 1.00 |
| PLpro | T819A | 3 | 2 | 1 | 2.7 | 1.6 | 1.0 | 1.00 |
| PLpro | A890D | 3 | 0 | 3 | 0.0 | 4.9 | 4.9 | 0.09 |
| PLpro | L1505C | 3 | 1 | 2 | 1.3 | 3.3 | 1.9 | 0.59 |
| PLpro | V1506F | 3 | 1 | 2 | 1.3 | 3.3 | 1.9 | 0.59 |
| PLpro | Y1535C | 3 | 2 | 1 | 2.7 | 1.6 | 1.0 | 1.00 |
| RdRP | L142F | 3 | 2 | 1 | 2.7 | 1.6 | 1.0 | 1.00 |
| S | S691T | 5 | 1 | 4 | 1.3 | 6.6 | 5.2 | 0.17 |
| S | N122Y | 4 | 3 | 1 | 4.0 | 1.6 | 2.4 | 0.63 |
| S | G700C | 4 | 1 | 3 | 1.3 | 4.9 | 3.6 | 0.33 |
| S | T95I | 3 | 1 | 2 | 1.3 | 3.3 | 1.9 | 0.59 |
| S | V143del | 3 | 1 | 2 | 1.3 | 3.3 | 1.9 | 0.59 |
| S | Y144del | 3 | 1 | 2 | 1.3 | 3.3 | 1.9 | 0.59 |
| S | R190S | 3 | 0 | 3 | 0.0 | 4.9 | 4.9 | 0.09 |
| S | N501Y | 3 | 1 | 2 | 1.3 | 3.3 | 1.9 | 0.59 |
| S | T547K | 3 | 1 | 2 | 1.3 | 3.3 | 1.9 | 0.59 |
| S | A570D | 3 | 1 | 2 | 1.3 | 3.3 | 1.9 | 0.59 |
| S | S686R | 3 | 1 | 2 | 1.3 | 3.3 | 1.9 | 0.59 |
| S | A694S | 3 | 0 | 3 | 0.0 | 4.9 | 4.9 | 0.09 |
| S | T859S | 3 | 2 | 1 | 2.7 | 1.6 | 1.0 | 1.00 |
| S | S982A | 3 | 1 | 2 | 1.3 | 3.3 | 1.9 | 0.59 |
| S | K1191N | 3 | 1 | 2 | 1.3 | 3.3 | 1.9 | 0.59 |

^a^ Percentage determined using the 75 participants in the SNG001 arm with sequencing available.

^b^ Percentage determined using the 61 participants in the Placebo arm with sequencing available.

**Supplemental Figure 1. Individuals treated with interferon-β1a and untreated individuals had similar rates of synonymous to nonsynonymous mutations.**

Synonymous average pairwise distance normalized to the day of last collected sequence (APD/day) (**A**), and dN/dS ratios during infection (**B**). Distributions of synonymous average pairwise distance and dN/dS ratios in the SNG001 and placebo arms are compared using two-sided Wilcoxon rank sum tests. Box plots depict median, interquartile range, and range.


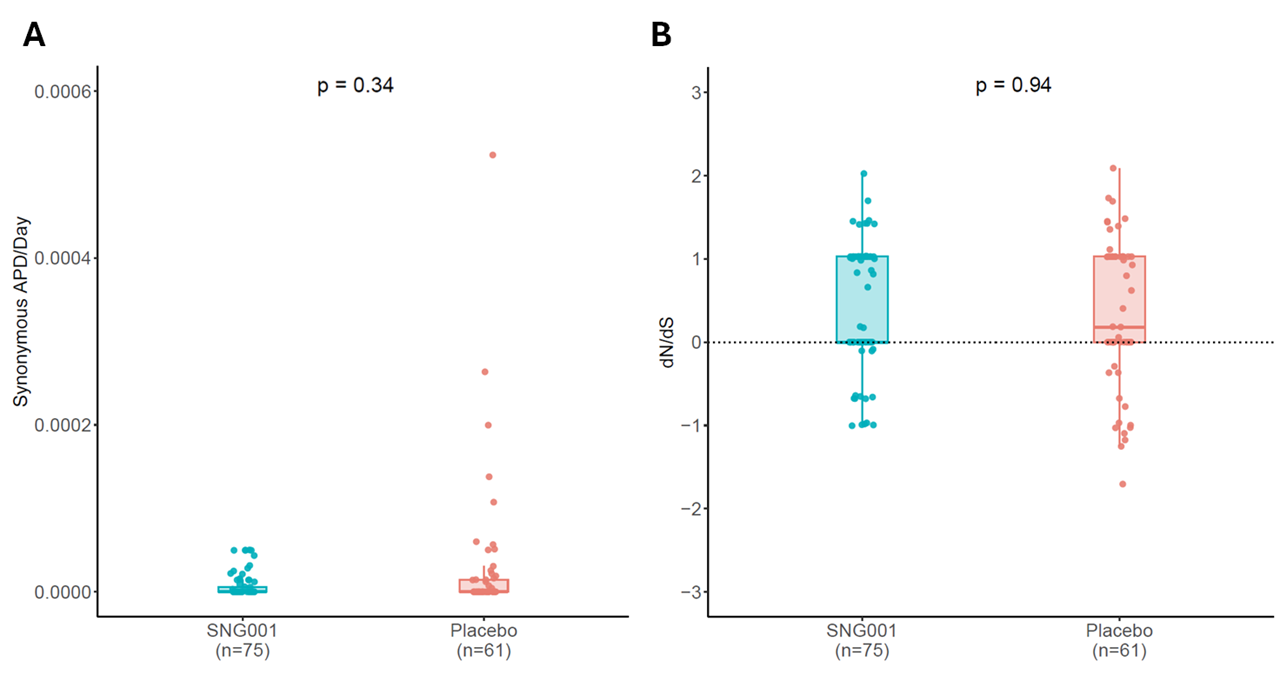


**Supplemental Figure 2. Four emerging nonsynonymous mutations occur more frequently in the placebo group participants.**

The percentage of the SNG001 and placebo groups developing emerging nonsynonymous mutations that occurred significantly more frequently in one study arm, without statistical correction for multiple comparisons. Abbreviations: ORF, open reading frame; nsp, non-structural protein; S, spike; RdRp, RNA-dependent RNA polymerase; GISAID, Global Initiative on Sharing All Influenza Data.


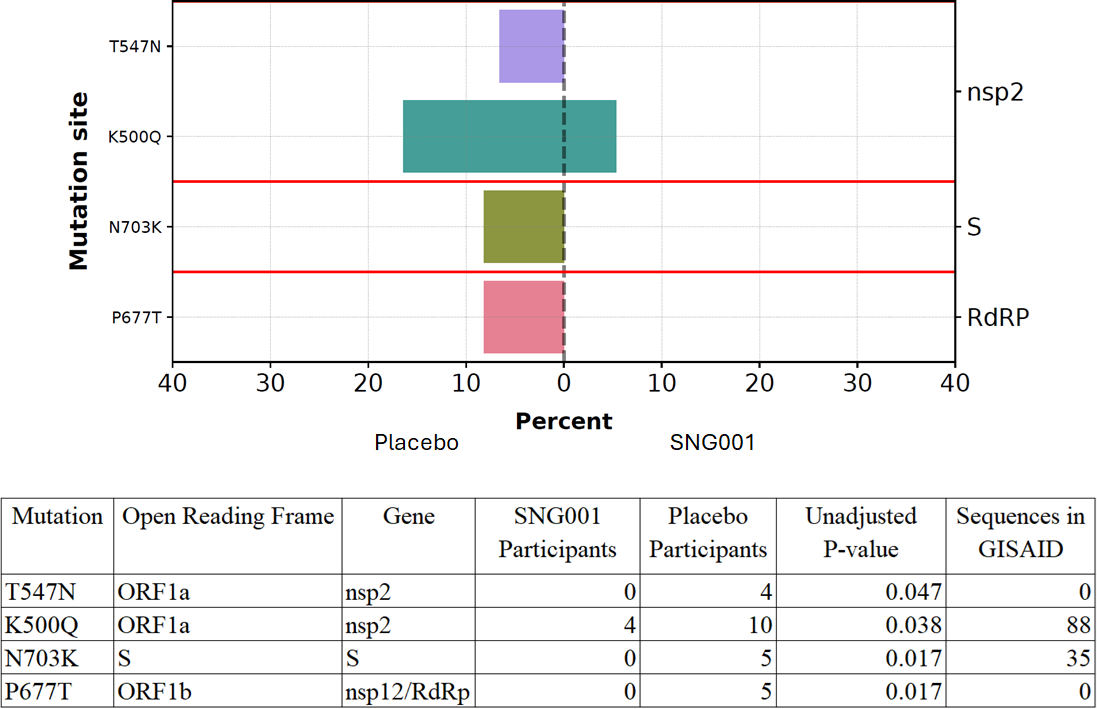

Supplement: Supplemental figures and tables — Fig. S1 and S2, and Tables S1 and S2. [file spectrum.00541-26-s0001.docx]
